# Retention of a Detached Robotic Scissors Tip Cover in the Abdominal Wall: A Case Report

**DOI:** 10.1111/ases.70242

**Published:** 2026-01-14

**Authors:** Masatsugu Kojima, Toru Miyake, Soichiro Tani, Keiji Muramoto, Yusuke Nishina, Sachiko Kaida, Katsushi Takebayashi, Hiromitsu Maehira, Reiko Otake, Haruki Mori, Nobuhito Nitta, Miyuki Kimura, Noritoshi Ushio, Tomoharu Shimizu, Masaji Tani

**Affiliations:** ^1^ Department of Surgery Shiga University of Medical Science Otsu Shiga Japan; ^2^ Department of Surgery Nagahama Red Cross Hospital Nagahama Shiga Japan; ^3^ Department of Nursing Shiga University of Medical Science Hospital Otsu Shiga Japan; ^4^ Department of Radiology Shiga University of Medical Science Hospital Otsu Shiga Japan; ^5^ Medical Safety Section Shiga University of Medical Science Hospital Otsu Shiga Japan

**Keywords:** computed tomography, foreign body, postoperative complication, robotic surgery, tip cover detachment

## Abstract

Robotic surgery has become increasingly widespread; however, device‐related complications specific to robotic platforms are rarely reported. We describe a case of rectal cancer treated with robotic‐assisted abdominoperineal resection using the da Vinci Surgical System. At the end of the procedure, the tip cover of the robotic scissors detached and was inadvertently retained within the abdominal wall. It was invisible on plain radiography but was detected on postoperative computed tomography as a cylindrical structure beneath the rectus abdominis muscle. The patient underwent reoperation to retrieve the tip cover and recovered uneventfully, being discharged without further complications. This case highlights a rare but important complication of robotic surgery—detachment and retention of the tip cover of robotic scissors in the abdominal wall. Because tip covers may not be reliably detected on plain radiography, computed tomography is crucial for their identification. Strict counting protocols and heightened awareness are essential to prevent such events.

## 1 Introduction

Robotic surgery has become increasingly widespread, offering advantages such as enhanced dexterity without hand tremors, multi‐jointed instrument articulation, and magnified three‐dimensional visualization. However, the expansion of robotic surgery has also revealed unique complications, and surgeons must remain vigilant to prevent them.

Robotics‐specific complications include instrument malfunctions, organ injuries associated with the absence of tactile feedback, and inadvertent patient injuries caused by robotic arms [1]. Furthermore, the risk of instrument breakage with intrabody detachment and retention should be considered [1, 2].

Here, we report a rare case in which the tip cover of robotic scissors detached intraoperatively and remained embedded within the abdominal wall. The clinical course is presented in detail, along with a discussion of the necessary measures to prevent recurrence of this complication.

## 2 Presentation of Case

**FIGURE 1:**
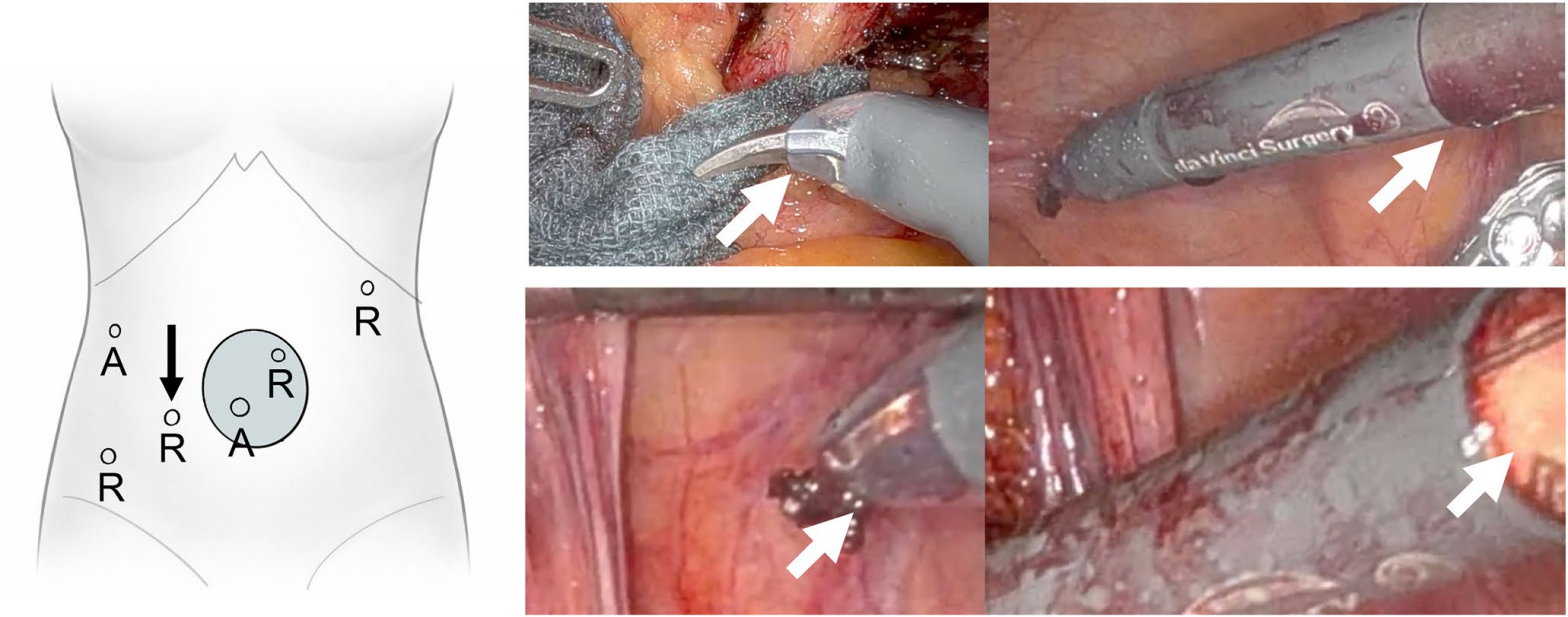
Port placement and intraoperative findings showing displacement of the robotic scissors tip cover. The left panel shows a schematic illustration of port placement for the robotic‐assisted abdominoperineal resection. Ports labeled “R” indicate those used for robotic instruments, whereas ports labeled “A” indicate those used for the assistant's instruments. At the umbilical site, a 4‐cm mini‐laparotomy was initially performed, and a multiport access device was placed; the ports were subsequently inserted through this device. The right panel shows intraoperative photographs of the robotic scissors. The upper image demonstrates the normal condition before tip cover displacement, in which the scissor tips are fully exposed and no gap is present between the tip cover and the instrument shaft. The lower image demonstrates the findings after displacement of the tip cover, showing reduced distal exposure of the scissor tips and a visible gap between the tip cover and the shaft, with exposure of the underlying orange‐colored component.

A patient with rectal cancer underwent robotic‐assisted abdominoperineal resection using the da Vinci Surgical System (Intuitive Surgical, Sunnyvale, CA, USA). The robotic scissors were introduced through the port indicated by the arrow in the left panel of Figure 1. Following completion of the final step of the robotic procedure, the assistant removed the scissors, and the scrub nurse received the instrument; however, neither recognized that the tip cover had detached, and the detachment was also not detected during the standard intraoperative post‐undocking instrument check. At our institution, the tip cover is routinely inspected and attached by the scrub nurse before the procedure, and no intraoperative reattachment was performed in this case. In retrospect, intraoperative images confirmed that the tip cover was initially positioned correctly but later shifted distally, reducing exposure of the scissor tips. A gap subsequently formed between the tip cover and the shaft, revealing the underlying orange‐colored component (right panel of Figure 1).

**FIGURE 2:**
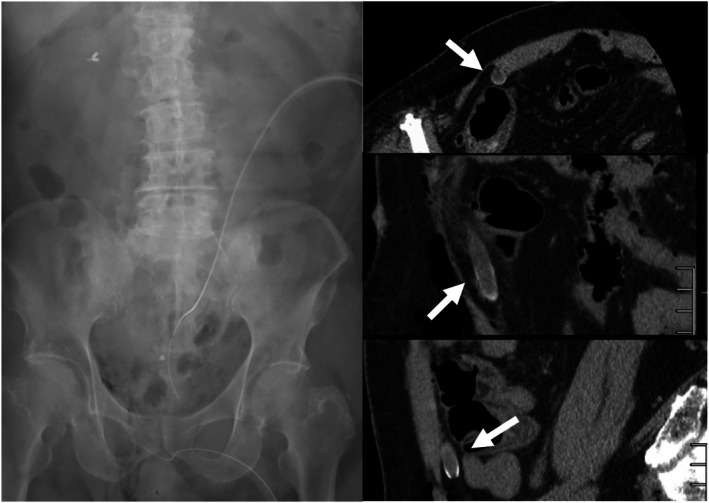
Postoperative imaging for detection of the retained foreign body (tip cover). An immediate postoperative abdominal radiograph obtained in the operating room failed to identify the foreign body, whereas computed tomography (axial, coronal, and sagittal views) successfully detected and localized it. The retained tip cover was located posterior to the right rectus abdominis muscle, above the ventral peritoneal fat, extending in a craniocaudal direction.

After the operation was completed, while he was already awake, extubated, and waiting to be transferred out, the scrub nurse noticed that the scissor tip cover was missing, raising suspicion of a retained foreign body. Because re‐examining the abdominal cavity laparoscopically at that point would have required re‐induction of anesthesia—and a negative laparoscopic inspection would have required awakening the patient again for CT—we elected to perform CT first. The CT revealed a cylindrical foreign body consistent with the missing tip cover, located beneath the right rectus abdominis muscle in the abdominal wall (Figure 2).

**FIGURE 3:**
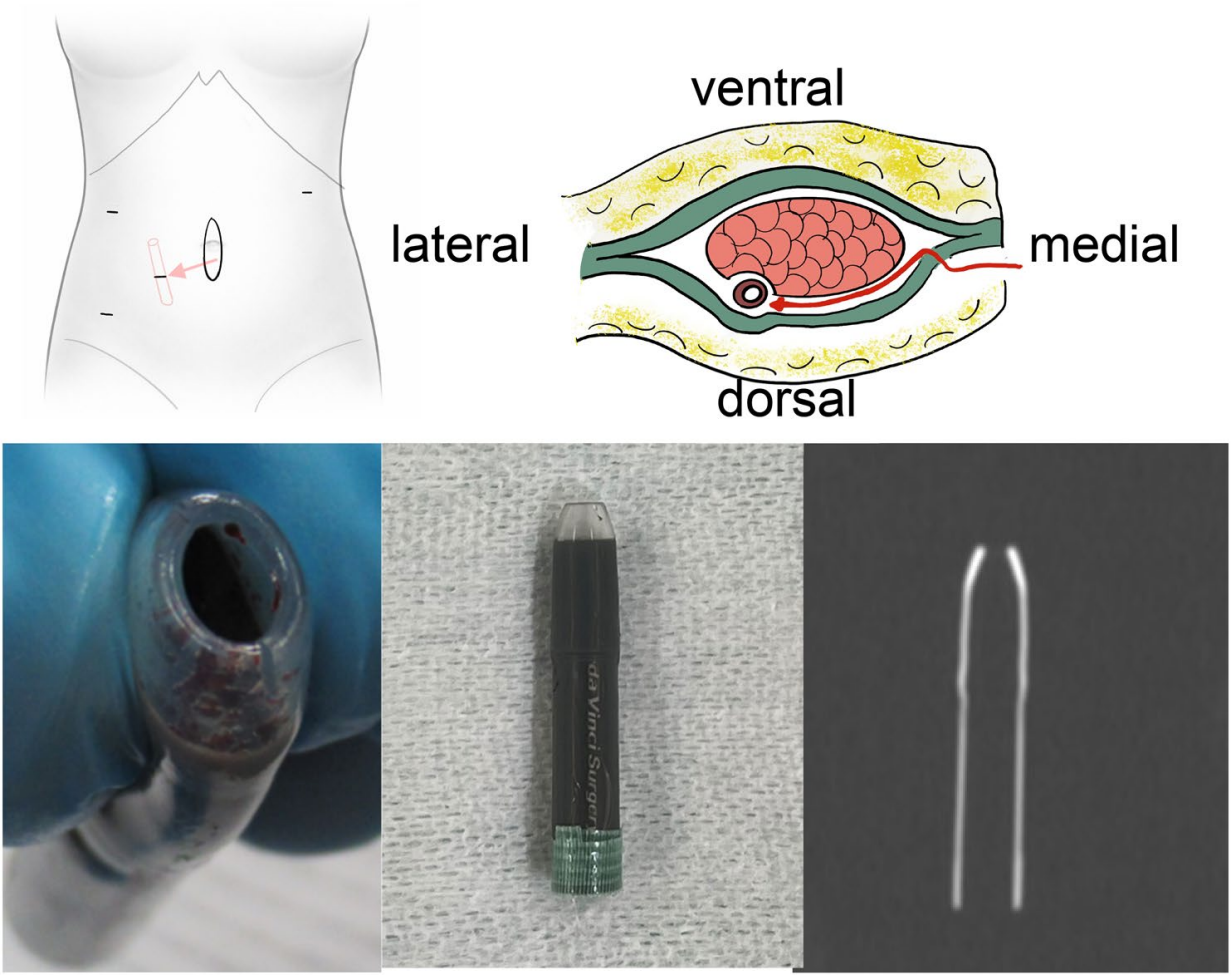
Localization and retrieval of the retained robotic tip cover with corresponding CT characteristics. The retained tip cover was approached through a midline incision (upper left part). After opening the posterior rectus sheath, the object was accessed from the dorsal side of the rectus abdominis muscle and retrieved (upper right part). The extracted tip cover showed a cylindrical hollow interior and consisted of three distinct components. Retrospective CT analysis demonstrated attenuation values of +150, −300, and −430 Hounsfield units, from the distal end proximally (lower part).

The patient was promptly returned to the operating room for reoperation. The umbilical midline incision was reopened, and palpation from the peritoneal side suggested the tip cover was retained within the abdominal wall. We approached the area behind the rectus abdominis muscle within the rectus sheath, identified the tip cover, and successfully retrieved it (Figure 3). The postoperative course was uneventful, and the patient was discharged without further complications.

## 3 Discussion

A large MAUDE database analysis reported that most robotic surgery–related adverse events stem from device or instrument malfunctions, including breakage, arcing, and unintended instrument operation [1]. These findings highlight the need to recognize platform‐specific failures. Intraoperative retention of surgical items occurs in 0.06%–0.11% of minimally invasive procedures [3]. Needle retention is the most frequently documented form; however, broken tips of suturing devices or disintegrated instrument components have also been reported [4, 5].

The tip cover of robotic scissors is designed to provide electrical insulation at the distal end of the instrument, thereby preventing arc‐related injuries during energy activation. Several adverse events involving tip covers have been reported, including perforation‐related arcing and junctional‐damage–related arcing causing injury to adjacent organs [6, 7]. Incorrect proximal placement of the tip cover toward the instrument arm has also been reported, resulting in impaired instrument mobility [8]. In our case, displacement occurred in the opposite direction, increasing the functional shaft diameter and potentially contributing to eventual detachment during instrument withdrawal. No previous reports have described a retained tip cover from robotic scissors. Detachable covers may be vulnerable to similar events, highlighting the need for awareness.

In this case, the tip cover became detached and was retained within the abdominal wall. The most plausible mechanism is as follows: after becoming partially dislodged, the tip cover likely increased the instrument's effective diameter, creating greater friction against the abdominal wall or port during withdrawal. This friction may have contributed to its detachment, and the detached tip cover was subsequently left within the abdominal wall during port removal.

Since the tip cover is non‐metallic, it could not be detected on plain radiography. In contrast, CT identified it based on its geometric configuration and subtle differences in attenuation values, underscoring the importance of CT evaluation when a foreign body not readily visible on plain radiography is suspected [9]. Retrospective CT analysis of the detached tip cover revealed that its three material components demonstrated distinct attenuation values: +150, −300, and −430 Hounsfield units (HU) from the distal end proximally (Figure 3). For comparison, skeletal muscle and fat tissue typically measure −29 to +150 and −190 to −30 HU, respectively [10]. These differences facilitated radiologic identification of the cover.

Failure to recognize the detachment intraoperatively may be attributed to several factors: neither the bedside assistant nor the scrub nurse noticed the event, and detachment occurred during removal of the final scissors, leaving no opportunity for re‐examination. Moreover, because the detached cover was located within the abdominal wall rather than the peritoneal cavity, it was not visible endoscopically. The tip cover was also not included among the items subjected to instrument counts.

To prevent similar incidents, several measures should be implemented. First, tip covers should be included in both preoperative and postoperative instrument counts. Second, awareness must be heightened not only among surgeons but also among assistants and scrub nurses regarding the potential for detachment of the tip covers of robotic scissors.

## Author Contributions

M.K. collected the clinical data and drafted the manuscript. All authors participated in the study design and coordination, contributed to drafting the manuscript, and critically revised it. All the authors have read and approved the final version of this manuscript.

## Funding

The authors have nothing to report.

## Ethics Statement

The authors have nothing to report.

## Consent

Written informed consent for the publication of this case report and accompanying images was obtained from the patient.

## Conflicts of Interest

The authors declare no conflicts of interest.

## Data Availability

Data sharing is not applicable to this article as no datasets were generated or analyzed during the current study.
